# Towards Clinically Relevant Oculomotor Biomarkers in Early Schizophrenia

**DOI:** 10.3389/fnbeh.2021.688683

**Published:** 2021-06-10

**Authors:** Fotios Athanasopoulos, Orionas-Vasilis Saprikis, Myrto Margeli, Christoph Klein, Nikolaos Smyrnis

**Affiliations:** ^1^2nd Department of Psychiatry, School of Medicine, National and Kapodistrian University of Athens, University General Hospital “ATTIKON”, Athens, Greece; ^2^Laboratory of Cognitive Neuroscience and Sensorimotor Control, University Mental Health, Neurosciences and Precision Medicine Research Institute “COSTAS STEFANIS”, Athens, Greece; ^3^Department of Child and Adolescent Psychiatry, Medical Faculty, University of Freiburg, Freiburg, Germany; ^4^Department of Child and Adolescent Psychiatry, Medical Faculty, University of Cologne, Cologne, Germany

**Keywords:** oculomotor, biomarker, schizophrenia, first episode psychosis, recent onset, ultra-high risk

## Abstract

In recent years, psychiatric research has focused on the evaluation and implementation of biomarkers in the clinical praxis. Oculomotor function deviances are among the most consistent and replicable cognitive deficits in schizophrenia and have been suggested as viable candidates for biomarkers. In this narrative review, we focus on oculomotor function in first-episode psychosis, recent onset schizophrenia as well as individuals at high risk for developing psychosis. We critically discuss the evidence for the possible utilization of oculomotor function measures as diagnostic, susceptibility, predictive, monitoring, and prognostic biomarkers for these conditions. Based on the current state of research we conclude that there are not sufficient data to unequivocally support the use of oculomotor function measures as biomarkers in schizophrenia.

## Introduction

Oculomotor paradigms have been extensively used over the last 50 years in the realm of psychiatric research, in order to assess the integrity of cognitive functions (Gooding and Tallent, 2001; Hutton, 2008; Canu et al., 2021) and evaluate pharmacological intervention (Karpouzian et al., 2019). Performance of these tasks provides a variety of parameters that can be objectively and quantitatively measured (Alexander and Martinez-Conde, 2019; Foulsham, 2019; Lencer et al., 2019; Pierce et al., 2019). Impairments in oculomotor tasks constitute some of the most replicated findings in studies of psychiatric disorders and especially in those of schizophrenia (Levy et al., 1993, 2000; Smyrnis et al., 2019). The expectation behind the use of oculomotor tasks is to associate specific patterns of performance abnormalities with particular clinical syndromes in order to provide tools for the diagnosis, prognosis, and assessment of the response to treatment (Smyrnis, 2008). This research then aspires to come up with reliable oculomotor biomarkers for psychiatric disorders to be used in clinical practice (Smyrnis et al., 2019).

One definition of a biological marker or biomarker in medicine (Califf, 2018) is the following: “a biomarker is a defined characteristic that is measured as an indicator of normal biological processes, pathogenic processes, or responses to an exposure or intervention”. This broad definition can be derived from molecular, histologic, radiographic, imaging, or physiologic characteristics. A number of different categories of biomarkers have been defined according to their assumed applications (Table 1).

**Table 1:** Categories of biomarkers and their definition.

Diagnostic	Detection of the presence of a disease or condition of interest
Monitoring	Measurement of treatment effectiveness
Predictive	Indicate the likelihood of benefiting from a specific therapy
Susceptibility/Risk	Transition from a healthy state to disease
Prognostic	Likelihood of patients’ overall outcome, regardless of therapy

Firstly, diagnostic biomarkers characterize the detection of the presence of a disease or condition of interest, or the identification of an individual with a subtype of the disease or condition (Califf, 2018). Monitoring biomarkers are used to determine the status of a medical condition, for evidence of exposure to a medical product or environmental agent, or to discern an effect of a medical product or biological agent used as treatment (Califf, 2018). A predictive biomarker is defined by the fact that the presence of the biomarker or change in the biomarker levels predicts that an individual or group of individuals is more likely to experience a favorable or unfavorable effect from the subjection to medical intervention or environmental agent (Califf, 2018). A prognostic biomarker is used to identify the likelihood of a clinical event, disease occurrence, or disease progression. It is crucial to distinguish prognostic biomarkers from susceptibility/risk biomarkers, which predict the transition from a healthy state to a disease state (Califf, 2018). Also, susceptibility/risk biomarkers are differentiated from predictive biomarkers, which, as mentioned before, pinpoint factors associated with the effect of intervention or exposure.

At this point, a note should be made on a similar yet distinct concept, that of the endophenotype. Both the terms biomarker and endophenotype have been used interchangeably in the psychiatric research literature (Prata et al., 2014). Endophenotypes reflect a linkage between a clinical phenotype and a specific genotype and a number of criteria have been proposed for their identification (for a review, see Gottesman and Gould, 2003).

Biomarkers have proven to be valuable tools in the clinical praxis of many medical specialties and are currently the focus of intensive research in medicine. However, for a number of reasons (for a critique, see Venkatasubramanian and Keshavan, 2016) their usage remains absent from the field of psychiatry. One of these reasons is the aetiological “blindness” of the current classification systems of psychiatric disorders, meaning that the underlying pathophysiology of psychiatric syndromes is not taken into account when defining nosological groups. This, in turn, leads to a situation in which one and the same nosological group is likely to be aetiologically heterogeneous while different nosological groups show aetiological overlap (Lee et al., 2019). This leads to a lack of diagnostic sensitivity and specificity of biomarkers, as will be explained below. The research on schizophrenia and oculomotor variables could provide a solution to this conundrum as there is a large body of work indicating robust oculomotor function deviances in schizophrenia (Table 2). The focus, in particular, should be towards the first presentation of the disorder, known as first episode psychosis (FEP), recent-onset schizophrenia, and also individuals at Ultra-High Risk (UHR) of schizophrenia. The ability to diagnose patients better in terms of underlying pathophysiological mechanisms, predict the conversion to psychosis risk for UHR individuals, or anticipate treatment effects in this group would be invaluable as they would set the course for a proper monitoring and therapeutic plan as early as possible in the disease process.

**Table 2:** Summary of deficits found in chronic schizophrenia and FEP patients.

Deficit	Studies in chronic schizophrenia patients	Studies in FEP patients
Decreased gain indicating a deficit in the control of smooth pursuit eye movements (SPEM)	Clementz et al. (1990), Abel et al. (1991), Iacono et al. (1992), Sweeney et al. (1993), Clementz et al. (1994), Levy et al. (1994), Sweeney et al. (1994), Clementz et al. (1996), Jacobsen et al. (1996), Thaker et al. (1996), Arolt et al. (1998), Levy et al. (2000), O’Driscoll and Callahan (2008), Lencer et al. (2010), Lencer et al. (2015), and Trillenberg et al. (2016)	Gooding et al. (1994), Hutton et al. (1998), Hutton et al. (2004), and Lencer et al. (2010)
Increased intra-subject variability of saccadic latencies indicating a deficit in cognitive stability	Smyrnis et al., 2009, Karantinos et al. (2014), Theleritis et al. (2014), and Damilou et al. (2016)	-
Increased spatial error and latency in memory-guided saccades indicating a deficit in volitional oculomotor control and spatial working memory	McDowell and Clementz (1996), Calkins et al. (2008), Camchong et al. (2008), Landgraf et al. (2008), and Caldani et al. (2016)	Reilly et al. (2006) and Reilly et al. (2007)
Higher error rate and latency in antisaccades indicating a deficit in volitional oculomotor control and inhibitory cognitive control	Clementz et al. (1994), Thaker et al. (1988), Sereno and Holzman (1995), Allen et al. (1996), Gooding et al. (1997), Radant et al. (1997, 2007), Crawford et al. (1998), Curtis et al. (2001), Reuter et al. (2005), Gooding and Basso (2008), Harris et al. (2009), Reilly et al. (2014), Millard et al. (2016), Caldani et al. (2016), and Kleineidam et al. (2019)	Hutton et al., 1998, Hutton et al. (2004), Harris et al. (2006), Nieman et al. (2007), De Wilde et al. (2008), Harris et al. (2009), Kirenskaya et al. (2017), and Kleineidam et al. (2019)
Lower responsive search score (RSS) indicating a deficit in selective attention and working memory	Kojima et al. (1990), Mather et al. (1992), Matsushima et al. (1992), Matsushima et al. (1998), Kojima et al. (2000), Kojima et al. (2001), Obayashi et al. (2001), Tonoya et al. (2002), Suzuki et al. (2009), Qiu et al. (2011), and Qiu et al. (2018)	Kojima et al. (1990)
Shorter scanpath length (in free view exploration tasks) indicating a deficit in volitional exploratory oculomotor behavior	Kojima et al. (1989), Kojima et al. (1990), Kojima et al. (1992), Obayashi et al. (2001), Ryu et al. (2001), Obayashi et al. (2003), Bestelmeyer et al. (2006), Benson et al. (2007), Takahashi et al. (2008), Beedie et al. (2012), and Sprenger et al. (2013)	Kojima et al. (1990)
For a detailed description of the deficits see Gooding and Basso (2008), Smyrnis et al. (2019), and Wolf et al. (2021)		

In the following, text we review the evidence for biomarker status for each one of the categories of biomarkers for oculomotor function indices in schizophrenia. We focus this narrative review only to early schizophrenia and in particular FEP and patients up to 2 years after onset (recent-onset). FEP is defined by three alternative definitions: (i) first treatment contact; (ii) duration of antipsychotic medication use; and (iii) duration of psychosis, with methodological limitations for each of these definitions (Breitborde et al., 2009). We also include studies investigating high-risk individuals. The At-Risk Mental State [also termed as the Clinical High-Risk state for psychosis (CHR-P), a synonym to UHR (Fusar-Poli et al., 2013)] is a prodromal phase of schizophrenia characterized by cognitive impairments, mood alterations, anxiety, attenuated psychotic symptoms and a decline in social and occupational functioning (Kahn et al., 2015). The literature search was conducted in the PubMed and Scopus databases. The studies that were taken into consideration were those that included FEP patients, patients with schizophrenia and duration up to 2 years and UHR individuals. Studies that included only chronic schizophrenia patients, patients with illness duration more than 3 years or did not mention illness duration at all were excluded. Key words for our search included the following: schizophrenia and oculomotor, schizophrenia and “eye movement”, schizophrenia and eye and biomarker, “first episode schizophrenia” and “eye movement”, “first episode schizophrenia” and biomarker, “recent onset schizophrenia” and “eye movement”.

## Diagnostic Biomarkers

Patients at the time of FEP exhibit a variety of deviances in oculomotor tasks when compared with healthy controls. The most robust and replicable finding is the elevated error rate in the antisaccade task (Hutton et al., 1998, 2004; Nieman et al., 2000, 2007; Harris et al., 2006; De Wilde et al., 2008; Harris et al., 2009; Kirenskaya et al., 2017; Kleineidam et al., 2019). Simultaneously, in the same task, an increased latency of the correct antisaccades has been found (Hutton et al., 2002; Harris et al., 2009; Kleineidam et al., 2019). In Smooth Pursuit Eye Movements (SPEM) patients show higher Root Mean Square Error (RMSE) values (Iacono et al., 1992), lower maintenance gain (both in the open-loop and the closed-loop conditions of pursuit), and more frequent catch-up saccades (Gooding et al., 1994; Hutton et al., 1998, 2004; Lencer et al., 2010). Studies involving memory-guided saccades have shown impairment in accuracy of remembered spatial locations (Reilly et al., 2006, 2007). Finally, prosaccade latencies have been found to be reduced in antipsychotic-naïve FEP patients in some studies (Reilly et al., 2005, 2006, 2007; Hill et al., 2008; Krebs et al., 2010), while unimpaired in others (Harris et al., 2009; see Smyrnis et al., 2019, for a review).

The usage of such deficits as diagnostic biomarkers has been repeatedly proposed (Lencer et al., 2015). However, two very important problems preclude the use of these indices as diagnostic biomarkers (Smyrnis et al., 2019). Firstly, the deficits in patients in such tasks are found on a group level and hardly ever hold for all members of the group, leading to sub-optimal diagnostic sensitivity. A possible compensatory approach could be the usage of scores arising from a combination of multiple variables (Morita et al., 2019). This was demonstrated impressively by Benson et al. (2012) who reached a 98.3% classification accuracy for schizophrenia vs. healthy controls on the basis of a brief assessment of fixation, smooth pursuit, and free viewing eye movements. Secondly, the other important requirement for a diagnostic biomarker, especially for early detection, is a high diagnostic specificity for the disease in question. Initial studies using qualitative measures of SPEM performance showed that deficits, although consistent, were not specific to schizophrenia, but were also detected in patients with major affective disorders such as bipolar disorder and major depression (Levy et al., 1993; Sweeney et al., 1994). Subsequently, studies using quantitative measures of pursuit performance such as closed-loop gain clarified that SPEM deficit is also prominent in patients with affective disorders (Abel et al., 1991; Kathmann et al., 2003; Brakemeier et al., 2019). Other studies employing the step-ramp pursuit task reported closed-loop and initial open-loop pursuit deficits both in schizophrenia and affective disorders (Sweeney et al., 1998, 1999; Lencer et al., 2004, 2010, 2015) while open-loop deficits were simultaneously found in schizophrenia and bipolar disorder patients (Trillenberg et al., 2016). Concerning the antisaccade task, studies with large sample sizes reported that patients with major depression (especially severe depression with psychotic characteristics) as well as patients with bipolar disorder (again, especially those with psychotic characteristics) also have elevated error rates compared to healthy controls (Tien et al., 1996; Sweeney et al., 1998; Gooding and Tallent, 2001; Martin et al., 2007; Harris et al., 2009). In the study of Harris et al. (2009), from a group of depression patients with and without psychotic features, it was shown that those with the psychotic features showed similar deficits as schizophrenic patients and patients with bipolar disorder. Reilly et al. (2014) compared the antisaccade error rate between groups of schizophrenia patients, schizoaffective patients, psychotic bipolar patients, and healthy controls. All patient’ groups presented with elevated error rates, and the schizophrenia patients also differed significantly from both schizoaffective and psychotic bipolar patients. In a study by Grootens et al. (2008), individuals with borderline personality disorder were included. It was shown that they produced more inhibition errors when compared with healthy controls, an increase which was more prominent in the individuals who also presented psychotic symptoms. The aforementioned studies have built a strong argument about increased antisaccade error rate being a trait of psychotic state, in and out of the context of schizophrenia (Gooding and Basso, 2008; Smyrnis et al., 2019). The genetic overlap between schizophrenia and bipolar disorder, but also between these and depression (Lee et al., 2019) may be involved in lowering the diagnostic specificity of the above oculomotor biomarkers.

Many studies have also reported elevated antisaccade error rates with (Lennertz et al., 2012) or without (Tien et al., 1992; Rosenberg et al., 1997) elevated antisaccade latency in patients with Obsessive-Compulsive Disorder (OCD). Damilou et al. (2016) compared the antisaccade task performance between OCD patients and schizophrenic patients and showed that both groups have similar increments in antisaccade error rate and antisaccade latency. Their only difference was in the variability of error prosaccade latencies, which were found increased only for the schizophrenia patients. This evidence indicates that saccadic eye movement abnormalities as measured with different variables are not restricted to patients with schizophrenia (Morita et al., 2019; Smyrnis et al., 2019) but are also observed in patients with other psychotic disorders and other psychiatric conditions such as OCD.

## Susceptibility Biomarkers

Susceptibility biomarkers are used to quantify the risk of transitioning from prodromal state to disease. Studies of UHR subjects could shed some light on the potential use of oculomotor functions as such. However, until now these studies have been very few and their findings are far from conclusive. van Tricht et al. (2010) showed that UHR participants exhibited higher rates of corrective and non-corrective saccades during a SPEM task. Caldani et al. (2017) also reported an increased number of intrusive saccades but only in their UHR subgroup with elevated neurological soft signs score. Nieman et al. (2007) reported a higher antisaccade error rate in UHR subjects as well as a trend towards comparatively higher baseline antisaccade error rates for those UHR participants who eventually transitioned into schizophrenia. Obyedkov et al. (2019) also reported an elevated antisaccade error rate in UHR. In contrast, Caldani et al. (2016) failed to find such a difference. Caldani et al. (2016) also used a memory-guided saccade task and reported an elevated error rate both for UHR and for biological healthy siblings of schizophrenia patients. In the study of Kleineidam et al. (2019), individuals at-risk mental state defined by cognitive basic symptoms as early (E-ARMS) or late at-risk mental state (L-ARMS) and patients with FEP, were studied using the prosaccade and antisaccade paradigm. L-ARMS but not E-ARMS participants exhibited elevated antisaccade latencies compared to controls. It was also reported that none of the variables analyzed (prosaccade and antisaccade latencies, antisaccade error rate, and correction rate) were predictive of conversion to psychosis within 2 years. Clearly, more studies with longitudinal design are needed in this field of research. Furthermore, the possibility that oculomotor variables could be used as predictors not only of conversion to psychosis but also of stability or de-escalation from the UHR status should also be considered.

## Monitoring/Predictive Biomarkers

Monitoring and predictive biomarkers can be used for monitoring the effects of treatment and predicting treatment outcomes. A few studies have used oculomotor paradigms in unmedicated or drug-naïve FEP. Hutton et al. (2001) examined whether smooth pursuit deficits in schizophrenia were comparable between first-episode and chronic schizophrenia patients who differed in their lifetime antipsychotic treatment administration and their antipsychotic treatment status at the time of testing. The chronic schizophrenia patients showed an elevated impairment in pursuit velocity gain compared to the FEP patients, and the degree of this effect was interceded by the effects of long–term antipsychotic treatment. Smooth pursuit velocity gain was found to be significantly better in chronic patients who were drug-free from antipsychotics for at least 6 months compared to those who did not stop their administration (Hutton et al., 2001).

The effect of medication in acute psychosis has been investigated by Hill et al. (2008), who found reduced latency of prosaccades in drug-naïve patients with FEP that was no longer present after 6 weeks of risperidone treatment. On the other hand, Keedy et al. (2014) report no effect of medication on prosaccade latency. With regard to the antisaccade paradigm, antipsychotic treatment (Hutton et al., 1998; Müller et al., 1999; Kallimani et al., 2009) and chronicity of the disease (Crawford et al., 1998; Curtis et al., 2001; Gooding et al., 2004) seem to have no impact on the core impairments. Increased frequency of antisaccade errors was present in FEP patients medicated at the time of testing (Hutton et al., 2002, 2004; Kleineidam et al., 2019) and treatment-naïve FEP patients (Harris et al., 2009). In one study, medicated and drug-naive FEP patients showed higher antisaccade error rates, but only drug- naïve patients also had an increased latency in initiating correct antisaccades (Hutton et al., 1998). Harris et al. (2006) reported a decrease in antisaccade error rates and latencies of correct antisaccades after treatment with haloperidol or risperidone. Studies comparing antisaccades early in the course of illness, prior and after treatment with atypical antipsychotics (Harris et al., 2006; Hill et al., 2008), noticed faster initiation of correct antisaccades but reported conflicting results on the error rate.

Some of the studies reported before, propose that selected oculomotor variables could be used as predictive/monitoring biomarkers. However, these studies examine only the medication effects on the oculomotor variables but not the overall relationship between treatment effects, changes in the oculomotor predictor variable, and changes in the symptomatology of the patient. To our knowledge, there are only four studies that have tried to explore this relationship. Gooding et al. (1994) studied FEP patients using a SPEM task and Root Mean Square Error (RMSE) as a measure of their performance. They reported that in their follow-up, after approximately 9.5 months, RMSE remained stable despite medication and clinical improvement. Obayashi et al. (2001) re-evaluated medicated schizophrenia patients (a mix of 24 FEP and 4 s-episode patients) after an average of 8 months in an exploratory eye movement paradigm. Dividing the patients into an improved and an unchanged group (based on their clinical improvement) they reported that exploratory eye movement variables [which included the number of eye fixations, mean eye scanning length (MESL), and RSS] did not change despite the improvement in clinical symptoms of the patients. Finally, Reilly et al. (2007) and Hill et al. (2008) failed to report any relationship between symptom change and oculomotor variables (including measures of visually guided saccades, antisaccades, and memory-guided saccades) change. They raised, however, an interesting point about utilizing those variables as monitoring agents for the cognitive adverse effects of antipsychotic treatment.

## Prognostic Biomarkers

It is well known that cognitive function deficits present in FEP and early schizophrenia are the most important predictors of the final outcome of the disorder (Díaz-Caneja et al., 2015; Suvisaari et al., 2018). Thus, the use of oculomotor function indices as prognostic biomarkers predicting the functional outcome of psychosis is probably the most promising area for the application of these measures in clinical practice. Nevertheless, only a few studies have reported such potential effects. RSS, of exploratory eye movement tasks, has been correlated both with negative symptoms (NS) in schizophrenia (blunted affect, emotional withdrawal; Kojima et al., 1990, 1992) as well as with hallucination severity (Qiu et al., 2018). Gaebel et al. (1987) reported a correlation between increased values of fixation parameters (mean duration of a single fixation as well as coefficient of variation) and negative symptoms. Increased RMSE in SPEM tasks was associated with generally impaired functioning (Katsanis et al., 1996). Obayashi et al. (2001) reported that mean eye scanning length (MESL) values were decreased in patients whose symptoms did not improve suggesting that MESL might be a sensitive indicator for monitoring chronicity in schizophrenia. Finally, the antisaccade error rate has been found higher in disorganized patients (DS) compared to patients with negative symptoms (NS) and patients with positive symptoms (PS), indicative of a correlation with specific clinical endophenotypes (Obyedkov et al., 2019).

Reviewing the available evidence it becomes clear that there is a great lack of studies evaluating the various oculomotor function indices as possible prognostic biomarkers. More longitudinal studies are needed to explore the prognostic value of oculomotor function deficits at FEP over the evolution of the disorder, the transition from the first episode to chronic schizophrenia, and the evolution of symptoms from positive symptoms and psychotic episodes to negative symptoms and functional and vocational impairment. Another important missing line of evidence is the investigation of oculomotor function deviances as prognostic biomarkers that would help the classification of patients between subgroups. Such biomarkers could provide critical clinical information for example dissociating the progression of FEP to either a debilitating chronic schizophrenia with prominent cognitive impairment and a poor functional outcome or a psychotic spectrum disorder with minimal cognitive impairment and a favorable functional outcome.

## Conclusion

In summary research of oculomotor deficits in early schizophrenia does not provide sufficient evidence for biomarker candidates. Individual variables lack both sensitivity and specificity as diagnostic biomarkers while there are not enough data for the use of oculomotor variables as susceptibility, predictive or prognostic biomarkers. Most of the studies do not set out with the question of whether an oculomotor variable can be used as a biomarker. They rather try to answer that a posteriori, an issue which seems to be common among the biomarker research in psychiatry (Prata et al., 2014). Longitudinal studies designed to investigate the effectiveness of oculomotor function measures as biomarkers combined with rigorous methodology are needed. Moreover, it is our belief that such studies should move away from the effort of detecting diagnostic oculomotor biomarkers in early schizophrenia and should focus on other types of biomarkers. Studies should be designed to explore the possibility that oculomotor function measures or combinations of them could be used as monitoring biomarkers for the effects of treatment and treatment outcome in early schizophrenia and FEP. As new therapeutic agents are being introduced in the treatment of cognitive deficits in schizophrenia oculomotor function measures could be target biomarkers assessing the effectiveness of such new treatments. Studies should be conducted addressing the relation of oculomotor function to patients’ symptomatology over the course of the disorder with the aim of predicting symptom progression especially in the realm of negative and cognitive symptoms and the transition to chronic schizophrenia with a poor functional outcome. Also, longitudinal studies will provide definite information for oculomotor function indices use as possible susceptibility/risk biomarkers in high-risk populations. The investigation and possible clinical implementation of oculomotor biomarkers is an intriguing prospect that should be introduced as an independent objective in oculomotor function research.

## Author Contributions

FA, MM, and O-VS did the literature search and wrote the original manuscript. NS and CK edited the final mansuscipt and conceived the idea for this review. All authors contributed to the article and approved the submitted version.

## Conflict of Interest

The authors declare that the research was conducted in the absence of any commercial or financial relationships that could be construed as a potential conflict of interest.
